# Ultraviolet Radiation and Basal Cell Carcinoma: An Environmental Perspective

**DOI:** 10.3389/fpubh.2021.666528

**Published:** 2021-07-22

**Authors:** Yan Teng, Yong Yu, Sujing Li, Youming Huang, Danfeng Xu, Xiaohua Tao, Yibin Fan

**Affiliations:** ^1^Department of Dermatology, Zhejiang Provincial People's Hospital, People's Hospital of Hangzhou Medical College, Hangzhou, China; ^2^Bengbu Medical College, Bengbu, China

**Keywords:** ultraviolet radiation, basal cell carcinoma, environmental, contributing factors, pathogenesis

## Abstract

Ultraviolet radiation (UVR) is a known carcinogen participated for the development of skin cancers. Solar UVR exposure, particularly ultraviolet B (UVB), is the mostly significant environmental risk factor for the occurrence and progress of basal cell carcinoma(BCC). Both cumulative and intermittent high-grade UVR exposure could promote the uncontrolled replication of skin cells. There are also exsiting other contributing environmental factors that combine with the UVR exposure to promote the development of BCC. DNA damage in formation of skin cancers is considered to be a result of UVR toxicity. It is UVR that could activate a series of oncogenes simultaneously inactivating tumor suppressor genes and aberrant proliferation and survival of keratinocytes that repair these damages. Furthermore, mounting evidence demonstrates that inflammatory responses of immune cells in the tumor microenvironment plays crucial role in the skin tumorigenesis as well. In this chapter, we will follow the function of UVR in the onset and development of BCC. We describe the factors that influence BCC induced by UVR, and also review the recent advances of pathogenesis of BCC induced by UVR from the genetic and inflammatory aspects.

## Introduction

Cutaneous cancer is the most common cancer type worldwide, and basal cell carcinoma (BCC) generally accounts for 75–80% of cases arising from the basal layer of the epidermis and its appendages (1–3). Most diagnosed patients are between 60 and 79 years old, and men are twice as likely to develop BCC. However, BCC incidence has tripled over the last 30 years; and recently, there has been a significant rise in younger individuals and women (4–6). The annual growth rate of BCC in Europe is approximately 5% over the recent decades. In the United States, the incidence rate increased by 2% annually, contributing to about 2–5 million patients with BCC receiving treatment every year (3, 7). Compared with Western countries, BCC incidence in the Asian population is 10- to 100-fold lower; but recently, there has been an increasing number of cases (8–10). Although, BCC rarely causes metastatic disease or death as the result of the extremely low mortality, it can result in significant morbidity because of its destructive local spread (11, 12). Ultraviolet radiation (UVR) is a known carcinogen that contributes to the development of cutaneous cancers containing both non-melanoma skin cancers (NMSCs) and malignant melanoma (MM). Solar UVR exposure, in particular ultraviolet B (UVB), is the most significant environmental risk factor for the occurrence and progress of BCC. Both cumulative and intermittent high-grade UVR exposures could promote the uncontrolled replication of skin cells. Although, other risk factors for skin carcinogenesis exist, UVR exposure has still been attributed to the development of nearly 90% of NMSCs, such as squamous cell carcinoma (SCC) and BCC (13, 14). DNA damage occurring during skin cancer formation is considered to be a result of UVR toxicity. UVR could activate a series of oncogenes while simultaneously inactivating tumor suppressor genes and aberrant proliferation and survival of keratinocytes that repair this damage. Furthermore, mounting evidence demonstrates that inflammatory responses of immune cells in the tumor microenvironment play a pivotal role in skin tumorigenesis (15). Therefore, environmental changes contributing to increased UV transmission have direct implications for human health. The public should be urged to use sunscreen and wear protective apparel to decrease BCC incidence.

In this review, we will assess the role of UVR in the onset and development of BCC. We describe the factors that influence BCC induced by UVR and review recent advances in BCC pathogenesis induced by UVR from genetic and inflammatory aspects. This is also a rare review to discuss the contributing factors associated with UVR-induced BCC and its specific pathogenesis. We searched associated studies using the following databases: Embase, Pubmed, Cochrane library, and Google Scholar. We conducted the literature by searching the Mesh terms denoting an exposure of interest (“UV rays,” “ultraviolet rays,” “UV radiation,” “ultraviolet radiation,” “UV,” “ultraviolet,” “environments,” “environmental impact,” and “environmental impacts”) and an outcome of interest (“basal cell carcinoma/cancer”). All studies included are published until December 30, 2020 with no language restrictions.

## The Factors Contributing to UVR-Induced BCC

The occurrence and development of BCC mainly depend on the interaction between general characteristics, such as genotypic and phenotypic features and subsequent environmental risk factor exposure. Therefore, as a primary risk factor, UVR could be combined with or influenced by other factors, such as general characteristics, UVR sources (such as sun exposure, tanning beds, and ultraviolet phototherapy), and other environmental factors (such as alcohol consumption, long-term chemical exposure, and photosensitive agents) to induce the onset of BCC.

## General Characteristics

The primary risk factor associated with BCC onset is directly related to the sun exposure habits of an individual or susceptibility to solar UVR (16). The result of a cross-sectional prevalence survey of white male watermen (*n* = 808) establishes the relationship between UVB exposure and BCC, SCC, and actinic keratosis (AK). It demonstrated that older age, childlike freckles, and blue eyes significantly enhanced the risk of skin tumors (17). In a prospective cohort, van Dam et al. (18) investigated the association of constitutional factors and sun exposure in BCC onset and development. They identified individuals with red hair, lightly pigmented eyes, northern European ancestry, and a predisposition to sunburn as likely to develop BCC. Another retrospective cohort study also revealed that multiple BCCs tended to be formed in the elderly and men. Patients with a history of BCC, type 1 or 2 skin, and chronic sun exposure (N500 weeks of sun exposure, a high photoaging score, and the presence of AK) have an increased risk of BCC (19). Several previous studies have demonstrated that obesity might decrease the risk of NMSC incidence (20–22), Chan et al. (23) designed a study to investigate whether the risk of NMSC of different weight levels is consistent with sun exposure risk. The results indicated that women with a BMI ≥ 25 kg/m^2^ or a WHR ≥.8 have a lower risk of NMSC. However, the influence of interactions with sun exposure should also be considered. This is because compared with the normal-weight group, hazard rates of the overweight group are higher when combined with increasing sun exposure time. Although, light exposure site is a common site for the development of BCC, it is not primarily associated with UV exposure. The simple theory that “More UV exposure, More skin cancer” could not perfectly explain the occurrence pattern of BCC. Heckmann et al. (24) investigated the correlation between anatomical prominences with peak UV exposure and frequencies and distinct histologic features of BCC. They found that the development of facial BCCs is poorly associated with the accumulative effects of UVR alone. Additionally, site-specific qualities, such as reduced tension and dermal thickness, could combine with UV exposure to promote the development of BCC. Additionally, there is no correlation between UV-exposed carcinoma and particular histologic features.

## Sources of UVR

Undoubtedly, sun exposure is the primary source of UVR associated with BCC onset. Artificial UVR exposure, namely, tanning beds, ultraviolet phototherapy, and arc welding has recently been reported to have a strong relationship with BCC onset.

## Sun Exposure

The result of a population-based, case-control study indicates that recreational sun exposure of children and adolescents increases risk, demonstrating that these life cycle stages are crucial to establishing the risk of developing BCC (25). The BCC risk is likely to depend on the nature and characteristics of sun exposure, such as pattern, timing, and amount. Previous studies have demonstrated that compared with cumulative, long-term UV exposure, intense intermittent exposure increases the risk of BCC (26, 27). Despite that, Iannacone et al. (28) studied the significance of sun exposure patterns (intermittent or continuous) and timing (childhood or adulthood) in BCC. Results suggested that sun exposure was associated with both the risk of BCC incidence regardless of the exposure pattern (intermittent or continuous). Therefore, the relationship between sun exposure pattern and BCC risk is still unproven and requires further research. UVR exposure dosage is positively linked with BCC risk, but after a certain amount of exposure, this effect level decreases or even disappears (29). Additionally, geographic variation is positively correlated with the incidence of BCC. The relationship between the incidence rate and proximity to the equator could be explained by higher UV exposure at lower latitudes, such as Hawaii, and higher UV exposure in higher latitudes, such as the Midwest (30, 31).

## Indoor Tanning

Indoor tanning first appeared in Western countries in the 1920's. Recently, this tanning trend has also become more prevalent among young people in China and other Asian countries who traditionally preferred lighter skin tones. Additionally, tanning services have increased, and online shops have begun to sell tanning products, such as tanning beds and tanning lamps. Indoor tanning equipment emits artificial UVR, which is one of the causes of skin cancer. Indoor tanning is also strongly correlated with BCCs of the trunk and extremities mainly exposed during tanning compared with head/neck lesions, which are only occasionally exposed to UVR to a considerable extent. The use of tanning beds has been correlated with an apparent increase in the development of NMSC (particularly early in life) (32, 33). A large case-control study revealed that indoor tanning was correlated with a 69% increase in the risk of early-onset BCC, and this was more evident in women, multiple BCCs, and BCCs located on the trunk and extremities. Previous studies have predicted that if individuals never tanned indoors, about a quarter of early-onset BCCs (or 43% in women) could be prevented (34). Several other studies also proved that indoor tanning is correlated with the incidence risk of early-onset BCCs (35). Compared with tanning bed use at the age of 25–35, the risk of BCC from using tanning beds during high school/college is significantly higher. There is also a dose-response link between sunbed use and the risk of skin cancers, particularly for BCC, and this relationship is more robust for younger patients exposed to sunbathing (36). Moreover, a meta-analysis showed that the relative risk of BCC development after indoor tanning before 25 years old was 1.4 (95% CI = 1.29–1.52). Compared with the control group, this translates to a 40% increase in risk. Studies have concluded that this risk will increase in a dose-dependent pattern with indoor tanning equipment usage (years) (37).

## Ultraviolet Phototherapy

Psoralen and ultraviolet A (PUVA) therapy and UVB therapy are both highly effective treatments for chronic cutaneous inflammatory diseases, in particular psoriasis. The BCC incidence related to ultraviolet phototherapy could be controversial. Previous studies have demonstrated that the BCC incidence rate among patients with psoriasis receiving a large number (>100–200) of PUVA treatments is significantly higher than expected, and this risk persists for some time after the discontinuation of PUVA therapy (38, 39). However, Stern et al. (40) suggested that substantial PUVA exposure only increases the risk of SCC, and even high-dose exposure to PUVA does not significantly increase BCC risk. Compared with the PUVA therapy, UVB (>300 treatments) is reported to be correlated with modest increases in the risk of developing BCC (41). However, the function of broadband UVB or narrowband UVB therapy in human skin carcinogenesis in psoriasis has not been clarified clearly (42, 43). A retrospective study showed that 80% of the statistical power in broadband UVB could detect six to seven times the increase in skin cancer, while in narrowband UVB, 83% of statistical power can detect five to six times the increase in skin cancer, and only one patient developed melanoma *in situ*. In this study, the tumor occurred within a year of phototherapy initiation. Hearn et al. (44) found no existing evident correlation between NB-UVB treatment and the incidence risk of BCC. Therefore, current studies do not offer strong evidence for increased BCC risk for patients treated with broadband and narrowband UVB phototherapy (45). Nevertheless, the concomitant potential risk of BCC should be considered when determining the risk of the therapy for long-term treatment of PUVA and UVB associated with the treatment for chronic inflammatory skin diseases (such as severe psoriasis).

## Arc Welding

Arc welding produces the full UVR spectrum, which may be a contributing cause of cutaneous cancer (46). Several case reports have reported the onset of BCC after several years of arc welding. Currie and Monk (47) reported five welders suffering from NMSC (average age of onset of 52 years). Three other case reports described welders developing BCC after exposure during unprotected arc welding (48). A study was conducted with a 25-year duration of systematic follow-up and eventually showed that long-term metal arc welding exposure might be associated with an increased BCC risk located exclusively at the neck. However, it could not provide evidence for the assumption that welding exposure enhances the risk in other locations (49).

## Other Environmental Factors

Although, UVR is the primary risk factor of BCC, recent evidence suggests that a small amount of UV exposure combined with other behavioral and/or environmental factors may lead to a higher incidence of BCC (50–52).

## Alcohol Consumption

Alcohol consumption is a well-known risk factor associated with various malignant tumors, namely, pharynx and larynx, esophagus, breast, prostate, pancreatic, and colon cancers (53–56). It has been observed that alcohol use can increase the prevalence of severe sunburn. It is hypothesized that the combination of alcohol consumption and UVR can enhance carcinogenicity in the skin *via* intermediate by-products or metabolites of alcohol (such as acetaldehyde), which can act as photosensitizers (57, 58). However, the association between alcohol consumption and the risk of developing BCC has been controversial. Although, two previous studies have reported a correlation between alcohol consumption and BCC risk, a subsequent study and several case–control studies did not show evident association (59–61). The result of a large prospective study conducted by Wu et al. has demonstrated that alcohol consumption is associated with an increased risk of BCC in both women and men. According to the result of a case–control study, alcohol consumption is generally associated with aggressive tumors. This may result from the modulation of the peritumoral micro-environment associated with alcohol consumption, which may be considered a contributing factor to the progression and malignant behavior of tumor cells.

## Citrus Products

Furocoumarins, a group of natural chemicals that are abundant in citrus products, have relatively high UV absorbance. Two large prospective cohort studies reported positive dose–response relationships between citrus consumption and the risk of BCC in two cohorts of men and women. Also, it is suggested that UVR could amply this association between the citrus consumption and the risk of BCC (62). In the European Prospective Investigation into Cancer and Nutrition cohort (EPIC) cohort study, Mahamat-Saleh et al. (63) also found that total citrus intake was associated with BCC risk. Specifically, they found that citrus juice intake was positively and linearly associated with BCC and mutagenic properties.

## Coffee Intake

Coffee intake has been demonstrated to have an anticarcinogenic potential in skin carcinogenesis. There is considerable and convincing experimental evidence that caffeine, which occurs naturally in seeds of the coffee plant, may have anti-proliferative effect *via* inducing apoptosis in UV-damaged keratinocytes *via* multiple pathways, such as the ataxia-telangiectasia and Rad3-related (ATR) kinase/ checkpoint kinase 1 (Chk1) pathway (64, 65). A review of the literature and meta-analysis conducted by Caini et al. (66) found that caffeinated coffee intake is moderately associated with a reduced risk of BCC development. However, the judgments on the strength of the evidence from the WCRF (World Cancer Research Fund International) regarding the influence of coffee on the BCC risk is evaluated to be limited suggestive. Increasing the number of randomized clinical trials is needed to verify the relationship.

## Vitamin D

Vitamin D has multiple functions for the human body *via* binding to vitamin D receptor (VDR) associated with cell growth, differentiation, apoptosis, and regulation of the immune system (67). Solar exposure is the major source of 25-hydroxyvitamin D3 (25-OH D3) synthesis. Vitamin D is known to have a protective effect against colon, breast, prostate cancers, and even NMSCs. It has been clarified that non-genomic pathways activated by vitamin D may have a protective role against DNA damage, which may contribute to the development of NMSCs (68). Ince et al. (69) found that maintaining the levels of 25-OH vitamin D3 more than 25 ng/ml in patients with an initial diagnosis of BCC can significantly decrease the recurrence rate. It could be a contradiction in terms that UV exposure has been recognized to be the predisposing factor of BCC. They think that patients with BCC should avoid sun exposure in the areas of high risk, but may expose other body areas without BCC during daylight for 10–15 min. The specific relationship between vitamin D and UV exposure in the development of BCC requests a long process of research.

## Long-Term Chemical Exposure

Arsenic is a non-metallic element that occurs naturally in air, soil, and water in organic and inorganic states. The organic state is non-toxic, while the inorganic state is toxic. Arsenic exposure elicits oxidative stress that causes DNA damage, genome instability, and telomere shortening (70–72). Epidemiological studies have shown that long-term exposure to arsenic increases cancer risk, such as bladder, lung, and kidney cancer (73, 74). Moreover, arsenic exposure has been widely involved in NMSC, and the strongest evidence comes from studies on people who drink highly polluted water (75, 76). Surdu et al. (77) conducted a study to evaluate airborne arsenic exposures at the workplace and quantify the relationship with NMSC. Eventually, they found no association between workplace exposure to arsenic and NMSC. However, it was suggested that women exposed to arsenic in the air with co-exposure to sunlight at work might be more susceptible to NMSC than those who are not exposed to the sun. In another multi-center case-control study, Srinivas et al. (78) observed that contrary to consistent reports about the association between increased telomere length and rising incidence risk of different cancers, among people exposed to arsenic, short telomeres are correlated with increased risk of BCC. Data analysis also showed that arsenic exposure could regulate the direction of the telomere length effect.

Radon is a type of radioisotope with a half-life of 3.8 days. Radon-222 gas originates from the radioactive decay of radium-226 and is present over the crust of the Earth and many building materials. The primary source of radon in buildings is the gas released from the ground, which enters a house through cracks in the basement. Radon gas accumulates indoors. The skin and lungs are both known to be the primary target organs.

Radon progeny exposure can result from alpha emitters, such as polonium-218 and polonium-214, which are classified as human carcinogens. The evidence of a link between radon and lung cancer risk is derived from studies on miners exposed to relatively high concentrations and exposure of the general population to radon indoors (79–82).

A large cohort study on a Danish population showed that long-term residential exposure might result in skin BCC development through detailed personal exposure assessment and control of several potential confounding factors. Investigators could not rule out confounding from sunlight exposure, nor could they conclude causality, because the correlation is stronger among people residing in apartments but not among those residing in single houses (83).

## Photosensitive Agents

The usage of photosensitizing medications has been identified to reduce UVR exposure that is likely to generate a sunburn-like erythema response, enhancing the risk of phototoxicity (84–86). A study (87) was conducted to assess the relationship between diuretic use and primary BCC, considering the history of sun exposure, constitutional characteristics, lifestyle factors, and geographically dispersed anthropometric measurements of individuals extensively exposed to ambient UVR. They found that among overweight participants, increased risk of BCC associated with diuretic use may be related to higher dosages, more extended periods of medication, decreased drug metabolism, or drug interaction. Tetracycline is a classical drug known to elicit photosensitivity, particularly phototoxic cutaneous disorders, and increase the susceptibility of the epidermis and dermis to UVR-induced damage. A prospective study demonstrated that tetracycline use is related to a mildly increased BCC risk but not to melanoma or SCC (88). Furocoumarins are natural chemicals that are abundant in certain plants, consisting of citrus products (89, 90). Animal model studies have shown the photocarcinogenic characteristics of furocoumarins (91). Wu et al. (62) designed a study to investigate the association between citrus consumption and BCC and SCC incidence risk. They found a positive correlation between BCC and SCC in two groups of men and women and called for studies to further understand the potential photocarcinogenesis related to dietary intake.

## The Pathogenesis of UVR-Induced BCC

Skin cancers caused by DNA damage are considered to be a direct result of UVR toxicity. UVR exposure can activate various oncogenes while inactivating tumor suppressor genes, leading to gene mutations, which induces the survival, and proliferation of keratinocytes, thereby, repairing this damage. Furthermore, increasing evidence shows that inflammatory responses of immune cells within a tumor microenvironment also significantly promote the onset of skin cancers.

## Genetic Mutations

Ultraviolet radiation exposure, particularly UVB, to some degree, could induce different categories of DNA damage, including cyclobutene pyrimidine dimers (CPD) and 6-4 photoproducts (6-4 PP), DNA strand breaks, and crosslinks. If not repaired entirely, this could transform into genetic mutations, ultimately resulting in skin carcinogenesis (92, 93). Here, we focus on some recently discovered genetic mutations involved in the onset and development of UVR-induced BCC.

## The Hedgehog Pathway-Associated Genes

As a highly conserved developmental pathway, the Hedgehog (Hh) pathway is responsible for various processes, such as organogenesis, stem cell maintenance, tissue repair, and regeneration (94). The Hh pathway is crucial for maintaining stem cell numbers and regulating hair follicle and sebaceous gland development in the skin. Abnormal activation of the Hh pathway induces various periods of tumorigenesis, such as onset, development, and recurrence (95, 96). The pathway could be separated into two categories, the canonical and non-canonical Hh pathways. The former includes some crucial components, consisting of Hh ligands as sonic Hh, Indian Hh, and Desert Hh; transmembrane receptor proteins PTCH1 and PTCH2, the G protein-coupled receptor-like protein SMO, and the GLI transcription factors 1, 2, and 3 (GLI1, GLI2, andGLI3) (97). When the Hh ligands bind to PTCH1, the pathway is activated, thereby, releasing PTCH-mediated SMO suppression of the primary cilium. SMO is in turn transported to the cilium, driving a signaling cascade and inducing the release of the GLI proteins, which are sequestered in the cytoplasm by several proteins, such as the suppressor of fused (SUFU). Then, GLI transcription factors enter the nucleus, activate the transcription of context-specific genes, and regulate self-renewal, cell fate, survival, and angiogenesis. Additionally, a feedback loop to automatically adjust the Hh signaling GLI1 has also been established to regulate the Hh signaling *via* PTCH1 modulation automatically (94, 98). Any level of genetic mutations in the Hh signaling pathway, such as PTCH1, SMO, and SUFU, will develop an increased expression of GLI1 (99). In BCC, upregulation of Hh signaling is proved to be the most significant pathogenic event (99, 100). Over 90% of BCCs have a deficiency of PTCH1 function by inactivating PTCH1 mutations and aberrant activation of SMO (101).

The *PTCH1* gene is mapped to 9q22.3, composed of 23 exons with a length of about 74 kb, encoding a 1,447 transmembrane glycoprotein (102). Somatic mutations of *PTCH1* range from 11–75%, which are mainly nonsense and splice site mutations through the entire length of the *PTCH1* gene, and there is no evidence of hot spot (103–105). About half of these mutations include the “UV-signature” C-T and tandem CC-TT transitions (106). However, the UVR source of *PTCH1* mutations is controversial, and other factors, such as oxidative stress, have been involved in the gene mutation (107, 108).

Ten to twenty percent of sporadic BCCs have the activating *SMO* mutations mainly manifested as missense mutations affecting codon 535 (109, 110). Functional studies of the W535L mutant showed that it is a constitutively active variant whose fundamental Hh activity is enhanced in the absence of a Hh ligand. Recent studies indicated that up to 8% of BCCs have dysfunctional SUFU mutations, such as both missense and non-sense mutations. This disrupts their binding to GLI, thereby, resulting in the activation of the constitutive pathway. Urman et al. reported a higher frequency of *SUFU* mutations (111), even though they are conceived to be a type of passenger mutation. Eventually, the homolog *PTCH2* gene has been muted in few sporadic BCCs, with 57% similarity to PTCH1 and acting as a receptor (112, 113).

## TP53

The second most common event related to the development of BCC is *TP53* gene inactivation. As a type of tumor suppressor gene, it participated in the activation of cell cycle arrest and programmed cell death. As the genome's guardian, *TP53* is stabilized by phosphorylation under pressure and alters the different downstream target gene expression categories, such as those that elicit cell cycle arrest (114). The inactivating *TP53* genetic alterations are detected among almost all skin carcinomas, considered to be an early event in skin carcinogenesis (115, 116). Most TP53 missense substitutions are located at the central DNA-binding core region (codons 102–292), including codons 177, 196, 245, 248, 278, and 282, which generate full-length protein function in skin cancers. Most of the *TP53* mutations in BCC are transition from C to T, and the frequency of double base changes from CC to TT is relatively high, which indicates alterations induced by UVR. Individuals who wear sunscreen have fewer TP53 mutations in BCCs compared with people who do not wear sunscreen (117).

## TERT

The *TERT* gene can maintain the length of telomere *via* encoding the catalytic reverse transcriptase subunit of telomerase. Increased telomerase activity is known to be one of the primary characteristics of human cancers, and the transcriptional mediation of the *TERT* gene is the main cause for its cancer-specific activation (118).

The *TERT* gene is located at chromosome 5p15.33. Its promoter region is considered to be the essential regulatory component of telomerase expression. TERT promoter mutations are frequently detected in various cancers such as skin and glioma (119, 120). They have been associated with increased TERT expression by recreating the binding sites for ETS/TCF transcription factors, higher telomere length, and poor prognostic factors. The fact that they are driving events in cancer development rather than passenger events is supported by the high recurrence, specificity, and functional acquisition of non-coding promoter TERT mutations. Several recent studies have investigated the role of the *TERT* promoter in BCCs and identified a high incidence of mutations. Most of these mutations have a UV-signature with C to T or CC to TT changes, which favors an etiologic role for UVR exposure (120–122).

## DPH3-OXNAD1 Bidirectional Promoter

Similar to the *TERT* gene, it is reported that in the bidirectional promoter of both *DPH3* and oxidoreductase NAD-binding domain containing 1 (*OXNAD1*) genes, recurrent mutations of non-coding sites close to the transcription start site are often present. *DPH3*, essential to the synthesis of diphthamide, is a modified histidine residue in the eukaryotic translation elongation factor 2, which helps keep the fidelity of translation. The silencing of *DPH3* can damage the *in vivo* deterioration of mouse melanoma cells. Its family member *DPH1* is also considered a tumor suppressor, necessary for the synthesis of diphthamide (123). Typical UVR mutations in the region of the *DPH3* promoter were recently shown to be ubiquitous in BCC (42%) (124). The adjacent site and binding motif of the ETS /TCF transcription factor were mutated at−8 and−9 bp of the DPH3 transcription start site.

## Ultraviolet Radiation-Induced Inflammatory Responses

Chronic inflammation participates significantly in all three periods necessary for BCC development, namely, initiation, promotion, and progression. The inflammatory responses induced by UVR contribute to increased blood flow and vascular permeability, leading to edematous erythema, thickening response, and cyclooxygenase-2 (COX-2) and prostaglandin (PG) metabolite activation. Inflammation recruits many leukocytes that secrete various pro-inflammatory cytokines at the UV-irradiated site and are considered necessary in the onset of the tumor. Additionally, various animal models and the effective use of anti-inflammatory chemotherapy agents all underline the significance of inflammation induced by UVR exposure in the onset and development of BCC (125–127).

## Nuclear Factor-Kappa B

Nuclear factor-kappa B (NF-kB), expressed in almost all types of cells, is a dimeric transcription factor that includes p50 and p65 Rel family proteins (128). Notably, it also functions in inflammatory response and cell proliferation, which are both associated with tumor onset. It is demonstrated that the constitutive expression of NF-kB is upregulated in a variety of tumor cells (129–131). UVR promotes the activation of IKKa, phosphorylation, and degradation of IkBa in epidermal keratinocytes. Interestingly, UVB sequentially mediates the activities of different subunits of NF-kB. NF-kB/p50 is downregulated in the early stage (6 h), and NF-kB/p65 is downregulated in the later stage (12 h) (132). Thus, UVB exposure is suggested to activate NF-kB, in turn leading to skin carcinogenesis. Weng et al. (133) found that NF-kB p65 might promote the highly aggressive type of BCC. It contributes to diagnosing malignant epidermal tumors, combined with TLR4 detection on epithelial cell membranes and p65 in epithelial cell nuclei. Tong and Wu (134) reported that activation of cNOS leads to the activation of NF-kB after UVB exposure. Continuous, rather than acute, suppression of IkB reduction and subsequent NF-kB activation is induced by the inhibition of cNOS.

## High-Mobility Group Box-1

High-mobility group box-1 is released into the cytoplasm and, in turn, extracellular matrix by interacting with the Toll-like receptors (TLRs) or receptor of advanced glycation end products (RAGE) to stimulate an inflammatory response. Recently Johnson and Wulff revealed that UVR induced the release of HMGB1 from *in vitro* keratinocytes, which is likely to be expressed in cutaneous tumors after recurrent and long-term exposure to UVR (134, 135). Similar to NF-kB, HMGB1 released by necrotic tumor cells was significantly expressed extracellularly in BCC. It is suggested that HMGB1 could be considered a potential prognostic indicator or treatment target for BCC treatment (136).

## Toll-Like Receptors

Toll-like receptors are expressed on various skin cells, such as keratinocytes and epidermis Langerhans cells, and function as a primary group of pattern recognition receptors activating skin immune responses (137). They mediate the pathogens and inflammatory response induced by potential endogenous molecules. Recent studies demonstrate that TLR activation contributes to the upregulation of host defense mechanisms and the upregulation of DNA repair genes, and increased functional DNA repair, thereby, providing an association between inflammatory response and DNA damage. DNA damage and repair induced by UVB radiation have been shown to involve TLR2, 3, 4, 7, 8, and 9 molecules. TLR7 is located in the endosome membrane and is highly expressed in BCC (138). Imiquimod, a kind of TLR7 agonist, is being extensively used topically nowadays to treat BCC (139). The results of animal research indicate that the mechanism of action for imiquimod is to enhance the expression of DNA repair genes and perform the functional repair of the DNA damage mechanism (140).

## Erbb2

As a proto-oncogene, Erbb2 (human epithelial growth factor receptor 2 (HER2)/neu) is activated in different types of cancer, related to invasive and treatment-resistant characteristics, which UVR exposure can activate. The expression of Erbb2 is located at both follicular and epidermal keratinocytes and acts in various crucial roles, such as regulating cell migration, differentiation, adhesion, inflammation, and angiogenesis after UVR exposure (141, 142). Suppression or deletion of Erbb2 inhibits cell proliferation, cell survival, and inflammation induced by UV. Recently, Rao et al. (143) indicated that it could accelerate skin carcinogenesis through the upregulation of ADAM12 (Disintegrin and metalloproteinase domain-containing protein 12). This demonstrated a new mechanism where the metastasis of UVR-induced BCC resulted from Erbb2.

## NLRP3

Nucleotide-binding domain, leucine-rich-repeat-containing family, pyrin domain-containing 3 (NLRP3) inflammasome is significant in innate immune responses by activating caspase-1 that contributes to the activation of pro-inflammatory cytokines, such as IL-1β and IL-18(144–146). Ahmad et al. (147) indicated that NLRP3 is found to be expressed in cells of human BCCs and is involved in the inflammatory response of BCC. In comparison, UVB exposure can inhibit Ca^2+^ mobilization by downregulating the expression of sarco/endoplasmic reticulum Ca^2+^-ATPase (SERCA2), which contributes to activation of the NLRP3 inflammasome.

## Cyclooxygenases

Increasing evidence demonstrates that COX are likely to participate in the formation of NMSC. It is known that COX mainly includes two isoforms, COX-1 and COX-2. Most cell types have a constitutive expression of COX-1, while various factors elicit the COX-2 expression. UVR exposure has been shown to enhance the expression of COX-2 in human skin. Furthermore, COX-1 and COX-2 are both demonstrated to have a role in BCC progression. Various pathways, such as AKT, p38, AMPK, and SIRT6, have been shown to regulate COX-2 associated with UVB (148, 149). Previous studies have shown that skin carcinogenesis is reduced by the suppression of p38a, AKT, and SIRT6, or activating AMPK (150–153).

Moreover, blocking the COX-2 expression may inhibit NMSC and is considered a functional chemopreventive agent for BCCs. However, it has been proved by animal studies that both selective COX-2 inhibitors like celecoxib and non-selective COX inhibitors (such as indomethacin and naproxen) can be regarded as effective agents to suppress BCCs induced by UVR (154–157).

## Interleukin-12

As a pleiotropic cytokine that participates in the inflammatory process, IL-12 consists of two subunits (p35 and p40), which have an antitumor function in various tumor models (158). It possesses an antitumor effect *via* repairing UVR-induced DNA damage in the form of cyclobutene pyrimidine dimmers (159–161). Meeran et al. (162) investigated the mechanism of antitumor activity of IL-12. They found that IL-12 deletion contributed to increased COX-2 expression and production of PGE2, along with upregulated inflammatory cytokines such as IL-1β, TNF-a, and IL-6. Infiltration of leukocytes, NF-kB activation, and cyclin D expression are induced *via* recombinant IL-12 before UVR exposure, confirming the function of IL-12 in the suppression of UVR-induced BCC.

## Conclusions

The incidence of BCC increases with age, while the etiology and mechanism of this disease are still not well-known. Its early diagnosis is difficult and often delayed. Furthermore, BCC rarely invades, metastasizes, or leads to death but contributes to widespread morbidity *via* tissue damage and local infiltration. Therefore, investigating the possible risk factors and the pathogenic mechanism is a worthwhile endeavor. As the primary risk factor in the etiology of BCC, excessive UVR exposure plays a crucial role in tumor-related gene mutation, microenvironment changes, and immune system disorders. The incidence of BCC induced by UVR is also influenced by several other factors, such as general characteristics, source of UVR, and other associated environmental factors. Additionally, an excessive amount of UVR exposure directly or indirectly induced DNA damage of the skin, contributing to mutations of an associated group of proto-oncogenes and tumor suppressor genes and alterations in the inflammation response, eventually leading to the onset and development of BCC. However, the understanding of UV-induced BCC is not comprehensive and complete. The association with its tumor-related genes, immune regulation, and inflammation response need to be further investigated to offer more effective and selective immunomodulatory strategies for patients with BCC that occurs in exposed areas.

## Author Contributions

YF: study concepts. XT and YT: study design. SL and YY: literature research. YT: manuscript preparation. YH: manuscript defnition of intellectual content. YT and DX: manuscript editing. XT: manuscript revision/review. YF: manuscript final version approval. All authors contributed to the article and approved the submitted version.

## Conflict of Interest

The authors declare that the research was conducted in the absence of any commercial or financial relationships that could be construed as a potential conflict of interest.

## References

[1] Szewczyk M Pazdrowski J Golusinski P Danczak-Pazdrowska A Łuczewski Ł Marszałek S. Basal cell carcinoma in farmers: an occupation group at high risk. Int Arch Occup Environ Health. (2015) 89:497–501. 10.1007/s00420-015-1088-0 26464316 PMC4786594

[2] Kim RH Armstrong AW. Non-melanoma skin cancer. Dermatol Clin. (2012) 30:125–39. 10.1016/j.det.2011.08.008 22117874

[3] Lomas A Leonardi-Bee J Bath-Hextall FA. A systemic review of worldwide incidence of non-melanoma skin cancer. Br J Dermatol. (2012) 166:1069–80. 10.1111/j.1365-2133.2012.10830.x 22251204

[4] Christenson L Borrowman T Vachon C Tollefson M Otley C Weaver A. Incidence of basal cell and squamous cell carcinomas in a population younger than 40 years. JAMA. (2005) 294:681–90. 10.1001/jama.294.6.681 16091570

[5] Birch-Johansen F Jensen A Mortensen L Olesen A Kjaer S. Trends in the incidence of non-melanoma skin cancer in Denmark 1978-2007: rapid incidence increase among young Danish women. Int J Cancer. (2010) 127:2190–8. 10.1002/ijc.25411 20473901

[6] Flohil S Seubring I Rossum M Coebergh J Vries E Nijsten T. Trends in basal cell carcinoma incidence rates: a 37-year Dutch observational study. J Invest Dermatol. (2012) 133:913–8. 10.1038/jid.2012.431 23190883

[7] Rogers HW Weinstock MA Feldman SR Coldiron BM. Incidence estimate of non-melanoma skin cancer (keratinocyte carcinomas) in the US population, 2012. JAMA Dermatol. (2015) 151:1081–6. 10.1001/jamadermatol.2015.1187 25928283

[8] Verkouteren J Ramdas KHR Wakkee M Nijsten T. Epidemiology of basal cell carcinoma: scholarly review. Br J Dermatol. (2017) 177:359–72. 10.1111/bjd.15321 28220485

[9] Rawashdeh M Matalka I. Basal cell carcinoma of the maxillofacial region: site distribution and incidence rates in Arab/Jordanians, 1991 to 2000. J Oral Maxillofac Surg. (2004) 62:145–9. 10.1016/j.joms.2003.04.009 14762745

[10] Abarca J Casiccia C. Skin cancer and ultraviolet-B radiation under the Antartic ozone hole: Southern Chile, 1987-2000. Photodermatol Photoimmunol Photomed. (2002) 18:294–302. 10.1034/j.1600-0781.2002.02782.x 12535025

[11] Marzuka A Book S. Basal cell carcinoma: pathogenesis, epidemiology, clinical features, diagnosis, histopathology, and management. Yale J Biol Med. (2015) 88:167–79. 26029015 PMC4445438

[12] Preston DS Stern RS. Non-melanoma cancers of the skin. N Engl J Med. (1992) 327:1649–62. 10.1056/NEJM199212033272307 1435901

[13] Marks R. The epidemiology of non-melanoma skin cancer: who, why and what can we do about it. J Dermatol. (2015) 22:853–7. 10.1111/j.1346-8138.1995.tb03935.x 8557858

[14] Pleasance ED Cheetham RK Stephens PJ McBride DJ Humphray SJ Greenman CD. A comprehensive catalogue of somatic mutations from a human cancer genome. Nature. (2010) 463:191–6. 10.1038/nature08658 20016485 PMC3145108

[15] Kim IY He YY. Ultraviolet radiation-induced non-melanoma skin cancer: regulation of DNA damage repair and inflammation. Genes Dis. (2014) 1:188–98. 10.1016/j.gendis.2014.08.005 25642450 PMC4307792

[16] Olsen C Wilson L Green A Bain C Fritschi L Whiteman D. Cancers in Australia attributable to exposure to solar ultraviolet radiation and prevented by regular sunscreen use. Aust Nz J Publ Heal. (2015) 39:471–6. 10.1111/1753-6405.12470 26437734 PMC4606762

[17] Vitasa BC Taylor HR Strickland PT Rosenthal FS West S Abbey H. Association of non-melanoma skin cancer and actinic keratosis with cumulative solar ultraviolet exposure in Maryland watermen. Cancer-Am Cancer Soc. (1990) 65:2811–7. 10.1002/1097-0142(19900615)65:12<2811::aid-cncr2820651234>3.0.co;2-u 2340474

[18] van Dam RM Huang Z Rimm EB Weinstock MA Donna S Colditz GA. Risk factors for basal cell carcinoma of the skin in men: results from the health professionals follow-up study. Am J Epidemiol. (1999) 150:459–68. 10.1093/oxfordjournals.aje.a010034 10472945

[19] Savas S Turgut Erdemir AV Koku Aksu AE Gurel MS Ozkur E. Clinical and prognostic factors in the development of basal cell carcinoma. Clin Dermatol. (2017) 35:616–23. 10.1016/j.clindermatol.2017.07.001 29191355

[20] Pothiawala S Qureshi AA Li Y Han J. Obesity and the incidence of skin cancer in US Caucasians. Cancer Causes Control. (2012) 23:717–26. 10.1007/s10552-012-9941-x 22450736 PMC3704194

[21] Tang JY Henderson MT Hernandez-Boussard T Kubo J Desai M Sims ST. Lower skin cancer risk in women with higher body mass index: the women's health initiative observational study. Cancer Epidemiol Biomarkers Prev. (2013) 22:2412–5. 10.1158/1055-9965.EPI-13-0647 24042260 PMC3880826

[22] Zhang Y Cartmel B Choy CC Molinaro AM Leffell DJ Bale AE. Body mass index, height and early-onset basal cell carcinoma in a case-control study. Cancer Epidemiol. (2017) 46:66–72. 10.1016/j.canep.2016.12.007 28039770 PMC5272867

[23] Chan A Noguti J Pak Y Qi L Caan B Going S. Interaction of body mass index or waist-to-hip ratio and sun exposure associated with non-melanoma skin cancer: a prospective study from the Women's Health Initiative. Cancer. (2018) 125:1133–42. 10.1002/cncr.31810 30548236

[24] Heckmann M Zogelmeier F Konz B. Frequency of Facial Basal Cell Carcinoma Does Not Correlate With Site-Specific UV Exposure. Arch Dermatol. (2002) 138:1494–7. 10.1001/archderm.138.11.1494 12437456

[25] Gallagher RP Hill GB Bajdik CD Fincham S Coldman AJ McLean DI. Sunlight exposure, pigmentary factors, and risk of nonmelanocytic skin cancer. Arch Dermatol. (1995) 131:157–63. 10.1001/archderm.1995.01690140041006 7857111

[26] Kricker A Armstrong BK English DR Heenan PJ. Does intermittent sun exposure cause basal cell carcinoma? A case-control study in Western Australia. Int J Cancer. (1995) 60:489–94. 10.1002/ijc.2910600411 7829262

[27] Rosso S Zanetti R Martinez C Tormo M Schraub S Sancho-Garnier H. The multicentre south European study 'Helios'. II: Different sun exposure patterns in the aetiology of basal cell and squamous cell carcinomas of the skin. Br J Cancer. (1996) 73:1447–54. 10.1038/bjc.1996.275 8645596 PMC2074492

[28] Iannacone MR Wang W Stockwell HG O Rourke K Giuliano AR Sondak VK. Patterns and timing of sunlight exposure and risk of basal cell and squamous cell carcinomas of the skin – a case–control study. BMC Cancer. (2012) 12:1–11. 10.1186/1471-2407-12-417 22994655 PMC3517361

[29] Kricker A Armstrong BK English DR Heenan PJ. A dose-response curve for sun exposure and basal cell carcinoma. Int J Cancer. (1995) 60:482–8. 10.1002/ijc.2910600410 7829261

[30] Chuang TY Popescu A Su WPD Chute CG. Basal cell carcinoma: a population-based incidence study in Rochester, Minnesota. J Am Acad Dermatol. (1990) 22:413–7. 2312827 10.1016/0190-9622(90)70056-n

[31] Reizner GT Chuang TY Elpern DJ Stone JL Farmer ER. Basal cell carcinoma in Kauai, Hawaii: the highest documented incidence in the United States. J Am Acad Dermatol. (1993) 29:184–9. 10.1016/0190-9622(93)70165-P 8335736

[32] Levine JA Sorace M Spencer J Siegel DM. The indoor UV tanning industry: a review of skin cancer risk, health benefit claims, and regulation. J Am Acad Dermatol. (2005) 53:1044. 10.1016/j.jaad.2005.07.066 16310065

[33] Light TIAF Cancer S. The association of use of sunbeds with cutaneous malignant melanoma and other skin cancers: a systematic review. Int J Cancer. (2007) 250:1116–22. 10.1002/ijc.22453 17131335

[34] Ferrucci LM Cartmel B Molinaro AM Leffell DJ Bale AE Mayne ST. Indoor tanning and risk of early-onset basal cell carcinoma. J Am Acad Dermatol. (2012) 67:552–62. 10.1016/j.jaad.2011.11.940 22153793 PMC3307842

[35] Karagas MR Zens MS Li Z Stukel TA Perry AE Gilbert-Diamond D. Early-Onset basal cell carcinoma and indoor tanning: a population-based study. Pediatrics. (2014) 134:e4–12. 10.1542/peds.2013-3559 24958589 PMC4067637

[36] Zhang M Qureshi AA Geller AC Frazier L Hunter DJ Han J. Use of tanning beds and incidence of skin cancer. J Clin Oncol. (2012) 30:1588–93. 10.1200/JCO.2011.39.3652 22370316 PMC3383111

[37] Wehner MR Shive ML Chren MM Han J Qureshi AA Linos E. Indoor tanning and non-melanoma skin cancer: systematic review and meta-analysis. BMJ. (2012) 345:e5909. 10.1136/bmj.e5909 23033409 PMC3462818

[38] Bruynzeel I Bergman W Hartevelt HM Kenter CCA Suurmond D. 'High single-dose' European PUVA regimen also causes an excess of non-melanoma skin cancer. Br J Dermatol. (1991) 124:49–55. 10.1111/j.1365-2133.1991.tb03281.x 1993145

[39] Nijsten TEC Stern RS. the increased risk of skin cancer is persistent after discontinuation of psoralen+ultraviolet a: a cohort study. J Invest Dermatol. (2003) 121:252–8. 10.1046/j.1523-1747.2003.12350.x 12880415

[40] Stern RS. The risk of squamous cell and basal cell cancer associated with psoralen and ultraviolet A?therapy: A?30-year prospective study. J Am Acad Dermatol. (2012) 66:562. 10.1016/j.jaad.2011.04.004 22264671

[41] Man I Crombie IK Dawe RS Ibbotson SH Ferguson J. The photocarcinogenic risk of narrowband UVB (TL-01) phototherapy: early follow-up data. Br J Dermatol. (2005) 152:755–7. 10.1111/j.1365-2133.2005.06537.x 15840109

[42] Patel RV Clark LN Lebwohl M Weinberg JM. Treatments for psoriasis and the risk of malignancy. J Am Acad Dermatol. (2009) 60:1017. 10.1016/j.jaad.2008.12.031 19344980

[43] Black RJ Gavin AT. Photocarcinogenic risk of narrowband ultraviolet B (TL-01) phototherapy: early follow-up data. Br J Dermatol. (2006) 154:566–7. 10.1111/j.1365-2133.2005.07085.x 16445801

[44] Hearn RMR Kerr AC Rahim KF Ferguson J Dawe RS. Incidence of skin cancers in 3867 patients treated with narrow-band ultraviolet B phototherapy. Br J Dermatol. (2008) 159:931–5. 10.1111/j.1365-2133.2008.08776.x 18834483

[45] Weischer M Blum A Eberhard F Cken MR Berneburg M. No evidence for increased skin cancer risk in psoriasis patients treated with broadband or narrowband UVB phototherapy: a first retrospective study. Acta Derm Venereol. (2004) 84:370–4. 10.1080/00015550410026948 15370703

[46] Bajdik C Gallagher R Astrakianakis G Hill G Fincham S Mclean D. Non-solar ultraviolet radiation and the risk of basal and squamous cell skin cancer. Br J Cancer. (1996) 73:1612–4. 10.1038/bjc.1996.303 8664139 PMC2074547

[47] Currie CL Monk BE. Welding and non-melanoma skin cancer. Clin Exp Dermatol. (2000) 25:28–9. 10.1046/j.1365-2230.2000.00565.x 10671966

[48] Dixon A. Arc welding and the risk of cancer. Aust Fam Physician. (2007) 36:255–6. 10.1186/1471-2296-8-18 17392941

[49] Heltoft KN Slagor RM Agner T Bonde JP. Metal arc welding and the risk of skin cancer. Int Arch Occup Environ Health. (2017) 90:873–81. 10.1007/s00420-017-1248-5 28766013 PMC5640727

[50] Gruijl D Frank R. Photocarcinogenesis: UVA vs. UVB radiation. Skin Pharmacol Physiol. (2002) 15:316–20. 10.1159/000064535 12239425

[51] Rao S Austin L Gao D Lu Y Phelps R Lebwohl M. The combination of Benzo[a]pyrene and ultraviolet a causes an in vivo time-related accumulation of DNA damage in mouse skin. Photochem Photobiol. (2003) 77:413–9. 10.1562/0031-8655(2003)077<0413:tcobau>2.0.co;2 12733653

[52] Rossman TG Uddin AN Burns FJ Bosland MC. Arsenite is a cocarcinogen with solar ultraviolet radiation for mouse skin: an animal model for arsenic carcinogenesis. Toxicol Appl Pharmacol. (2001) 176:64–71. 10.1006/taap.2001.9277 11578149

[53] Chen WY Rosner B Hankinson SE Colditz GA Willett WC. Moderate alcohol consumption during adult life, drinking patterns, and breast cancer risk. JAMA. (2011) 306:1884–90. 10.1001/jama.2011.1590 22045766 PMC3292347

[54] Gong Z Kristal AR Schenk JM Tangen CM Goodman PJ Thompson IM. Alcohol consumption, finasteride, and prostate cancer risk: results from the prostate cancer prevention trial. Cancer. (2009) 115:3661–9. 10.1002/cncr.24423 19598210 PMC2739798

[55] Jiao L Silverman DT Schairer C Thiébaut AC Hollenbeck AR Leitzmann MF. Alcohol use and risk of pancreatic cancer: the NIH-AARP diet and health study. Am J Epidemiol. (2009) 169:1043–51. 10.1093/aje/kwp034 19299403 PMC2727241

[56] Cho E Smith-Warner S Ritz J Brandt P Colditz G Folsom A. Alcohol intake and colorectal cancer: a pooled analysis of 8 cohort studies. Ann Intern Med. (2004) 140:603–13. 10.7326/0003-4819-140-8-200404200-00007 15096331

[57] Warthan MM Sewell DS Marlow RA Warthan ML Wagner RF. The economic impact of acute sunburn. Arch Dermatol. (2003) 139:1003–6. 10.1001/archderm.139.8.1003 12925386

[58] Saladi RN Nektalova T Fox JL. Induction of skin carcinogenicity by alcohol and ultraviolet light. Clin Exp Dermatol. (2010) 35:7–11. 10.1111/j.1365-2230.2009.03465.x 19778305

[59] Ansems TMR Van DPJC Hughes MC Ibiebele T Marks GC Green AC. Alcohol intake and risk of skin cancer: a prospective study. Eur J Clin Nutr. (2007) 62:162–70. 10.1038/sj.ejcn.1602717 17392700

[60] Milán T Verkasalo PK Kaprio J Koskenvuo M. Lifestyle differences in twin pairs discordant for basal cell carcinoma of the skin. Brit J Dermatol. (2003) 149:115–23. 10.1046/j.1365-2133.2003.05352.x 12890204

[61] Sahl WJ Glore S Garrison P Oakleaf K Johnson SD. Basal cell carcinoma and lifestyle characteristics. Int J Dermatol. (1995) 34:398–402. 10.1111/j.1365-4362.1995.tb04440.x 7657438

[62] Wu S Eunyoung C Diane F Li WQ Qi S Han J. Citrus consumption and risk of basal cell carcinoma and squamous cell carcinoma of the skin. Carcinogenesis. (2015) 36:1162–8. 10.1093/carcin/bgv109 26224304 PMC4834848

[63] Mahamat-Saleh Y Cervenka I Al-Rahmoun M Mancini FR Kvaskoff M. Citrus intake and risk of skin cancer in the European Prospective Investigation into Cancer and nutrition cohort (EPIC). Eur J Epidemiol. (2020) 35:1057–67. 10.1007/s10654-020-00666-9 32710289

[64] Vaseghi G Haghjoo-Javanmard S Naderi J Eshraghi A Mansourian M. Coffee consumption and risk of non-melanoma skin cancer: a dose-response meta-analysis. Eur J Cancer Prev. (2016) 27:1. 10.1097/CEJ.0000000000000322 27902644

[65] Song F Qureshi AA Han J. Increased caffeine intake is associated with reduced risk of basal cell carcinoma of the skin. Cancer Res. (2012) 72:3282–9. 10.1158/0008-5472.CAN-11-3511 22752299

[66] Caini S Cattaruzza MS Bendinelli B Tosti G Masala G Gnagnarella P. Coffee, tea, and caffeine intake and the risk of non-melanoma skin cancer: a review of the literature and meta-analysis. Eur J Nutr. (2017) 56:1–12. 10.1007/s00394-016-1253-6 27388462

[67] Giovannucci E. Vitamin D and cancer incidence in the harvard cohorts. Ann Epidemiol. (2009) 19:84–8. 10.1016/j.annepidem.2007.12.002 18291673

[68] Deeb KK Trump DL Johnson CS. Vitamin D signalling pathways in cancer: potential for anticancer therapeutics. Nat Rev Cancer. (2007) 7:684–700. 10.1038/nrc2196 17721433

[69] Ince B Yildirim M Dadaci M. Assessing the effect of vitamin d replacement on basal cell carcinoma occurrence and recurrence rates in patients with vitamin D deficiency. Hormones and Cancer. (2019) 10:145–9. 10.1007/s12672-019-00365-2 31254251 PMC10355704

[70] Maloney M. Arsenic in dermatology. Dermatol Surg. (2013) 22:301–4. 10.1111/j.1524-4725.1996.tb00322.x 8599743

[71] Bhattacharjee P Banerjee M Giri AK. Role of genomic instability in arsenic-induced carcinogenicity. A review. Environ Int. (2013) 53:29–40. 10.1016/j.envint.2012.12.004 23314041

[72] Fouquerel E Lormand J Bose A Lee HT Kim GS Li J. Oxidative guanine base damage regulates human telomerase activity. Nat Struct Mol Biol. (2016) 23:1092–100. 10.1038/nsmb.3319 27820808 PMC5140714

[73] Steinmaus C Ferreccio C Acevedo J Yuan Y Liaw J Durán V. Increased Lung and Bladder Cancer Incidence in Adults after In Utero and Early-Life Arsenic Exposure. Cancer Epidem Biomar. (2014) 23:1529–38. 10.1158/1055-9965.EPI-14-0059 24859871 PMC4344186

[74] Leonardi G Vahter M Clemens F Goessler W Gurzau E Hemminki K. Inorganic arsenic and basal cell carcinoma in areas of Hungary, Romania, and Slovakia: a case–control study. Environ Health Persp. (2012) 120:721–6. 10.1289/ehp.1103534 22436128 PMC3346769

[75] Smith AH Steinmaus CM. Arsenic in drinking water. J Environ Health. (1999) 9:36. 10.1016/S1074-9098(02)00298-8

[76] IARC Working Group on the Evaluation of Carcinogenic Risks to Humans. Some Drinking-Water Disinfectants and Contaminants, Including Arsenic. IARC Monographs on the Evaluation of Carcinogenic Risks to Humans. Vol. 84 (2004). p. 269–477. PMC5220262 15645578

[77] Surdu S Fitzgerald EF Bloom MS Boscoe FP Fletcher T. Occupational exposure to arsenic and risk of non-melanoma skin cancer in a multinational European study. Int J Cancer. (2013) 133:2182–91. 10.1002/ijc.28216 23595521

[78] Srinivas N Rachakonda S Hielscher T Calderazzo S Rudnai P Gurzau E. Telomere length, arsenic exposure and risk of basal cell carcinoma of skin. Carcinogenesis. (2019):6. 10.1101/465732 30874287

[79] Kendall GM Smith TJ. Doses to organs and tissues from radon and its decay products. J Radiol Prot. (2002) 22:389–406. 10.1088/0952-4746/22/4/304 12546226

[80] Darby S Hill D Doll R. Radon: a likely carcinogen at all exposures. Ann Oncol. (2001) 12:1341–51. 10.1023/A:1012518223463 11762803

[81] Cancer IAFR. Man-made mineral fibres and radon. J Clin Pathol. (1988) 42:1315–6. 10.1136/jcp.42.12.1315-d

[82] Darby S Hill D Auvinen A Barros-Dios J Baysson H Bochicchsio F. Radon in homes and risk of lung cancer: collaborative analysis of individual data from 13 European case-control studies. BMJ. (2005) 330:223. 10.1136/bmj.38308.477650.63 15613366 PMC546066

[83] Bräuner EV Loft S Sørensen M Jensen A Andersen CE Ulbak K. Residential radon exposure and skin cancer incidence in a prospective danish cohort. PLoS ONE. (2015) 10:e0135642. 10.1371/journal.pone.0135642 26274607 PMC4537191

[84] Glatz M Hofbauer GFL. Phototoxic and photoallergic cutaneous drug reactions. Chem Immunol Allergy. (2012) 97:167–79. 10.1159/000335630 22613861

[85] Moore D. Drug-induced cutaneous photosensitivity: incidence, mechanism, prevention and management. Drug Saf. (2002) 25:345–72. 10.2165/00002018-200225050-00004 12020173

[86] Stern R. Photocarcinogenicity of drugs. Toxicol Lett. (1999) 102–3:389–92. 10.1016/S0378-4274(98)00237-9 10022284

[87] McDonald E Freedman MD Alexander HB Doody MM Tucker MA Linet MS. Prescription diuretic use and risk of basal cell carcinoma in the nationwide U.S. radiologic technologists cohort. Cancer Epidemiol Biomarkers Prev. (2014) 23:1539–45. 10.1158/1055-9965.EPI-14-0251 24812037 PMC4119543

[88] Li W Drucker A Cho E Laden F Vopham T Li S. Tetracycline use and risk of incident skin cancer: a prospective study. Brit J Cancer. (2017) 118:294–8. 10.1038/bjc.2017.378 29073637 PMC5785738

[89] Dugo P Piperno A Romeo R Cambria M Russo M Carnovale C. Determination of oxygen heterocyclic components in citrus products by HPLC with UV detection. J Agr Food Chem. (2009) 57:6543–51. 10.1021/jf901209r 19722564

[90] Frérot E Decorzant E. Quantification of total furocoumarins in citrus oils by HPLC coupled with UV, fluorescence, and mass detection. J Agr Food Chem. (2004) 52:6879–86. 10.1021/jf040164p 15537290

[91] Wu S Han J Feskanich D Cho E Stampfer M Willett W. Citrus consumption and risk of cutaneous malignant melanoma. J Clin Oncol. (2015) 33:2500–8. 10.1200/JCO.2014.57.4111 26124488 PMC4979231

[92] Santoianni P Nino M. Sun light and skin cancer risk factors. Giorn Ital Dermat V. (2003) 138:455–64.

[93] Brash D Rudolph JA Simon JA Lin A McKenna G Baden H. A role for sunlight in skin cancer: UV-induced p53 mutations in squamous cell carcinoma. Proc Natl Acad Sci U S A. (1991) 88:10124–8. 10.1073/pnas.88.22.10124 1946433 PMC52880

[94] Katoh Y Katoh M. Hedgehog target genes: mechanisms of carcinogenesis induced by aberrant hedgehog signaling activation. Curr Mol Med. (2009) 9:873–86. 10.2174/156652409789105570 19860666

[95] Daya-Grosjean L Couvé-Privat S. Sonic hedgehog signaling in basal cell carcinomas. Cancer Lett. (2005) 225:192. 10.1016/j.canlet.2004.10.003 15978322

[96] Justilien V Fields AP. Molecular pathways: novel approaches for improved therapeutic targeting of hedgehog signaling in cancer stem cells. Clin Cancer Res. (2015) 21:505–13. 10.1158/1078-0432.CCR-14-0507 25646180 PMC4316382

[97] Briscoe J Thérond P. The mechanisms of Hedgehog signaling and its roles in development and disease. Nature reviews. Mol Cell Biol. (2013) 14:416–29. 10.1038/nrm3598 23719536

[98] Pellegrini C Maturo MG Di Nardo L Ciciarelli V García-Rodrigo C Fargnoli MC. Understanding the molecular genetics of basal cell carcinoma. Int J Mol Sci. (2017) 18:2485. 10.3390/ijms18112485 29165358 PMC5713451

[99] Bangs F Anderson KV. Primary cilia and mammalian hedgehog signaling. Csh Perspect Biol. (2016) 9:a28175. 10.1101/cshperspect.a028175 27881449 PMC5411695

[100] Bale AE Kuan-ping Y. The hedgehog pathway and basal cell carcinomas. Hum Mol Genet. (2001) 10:757. 10.1093/hmg/10.7.757 11257109

[101] Athar M Li C Kim A Spiegelman V Bickers D. Sonic hedgehog signaling in basal cell nevus syndrome. Cancer Res. (2014) 74:4953–4. 10.1158/0008-5472.CAN-14-1666 25172843 PMC4167483

[102] Teh M Blaydon D Chaplin T Foot N Skoulakis S Raghavan M. Genomewide single nucleotide polymorphism microarray mapping in basal cell carcinomas unveils uniparental disomy as a key somatic event. Cancer Res. (2005) 65:8597–603. 10.1158/0008-5472.CAN-05-0842 16204023

[103] Reifenberger J Wolter M Knobbe CB Köhler B Reifenberger G. Somatic mutations in the PTCH, SMOH, SUFUH, and TP53 genes in sporadic basal cell carcinomas. Br J Dermatol. (2005) 152:43–51. 10.1111/j.1365-2133.2005.06353.x 15656799

[104] Santos DCC Zaphiropoulos PG Neto CF Pimentel ERA Ruiz IRG. PTCH1 gene mutations in exon 17 and loss of heterozygosity on D9S180 microsatellite in sporadic and inherited human basal cell carcinomas. Int J Dermatol. (2011) 50:838–43. 10.1111/j.1365-4632.2010.04866.x 21699520

[105] Huang YS Bu DF Li XY Ma ZH Li H. Unique features of PTCH1 mutation spectrum in Chinese sporadic basal cell carcinoma. J Eur Acad Dermatol Venereol. (2012) 27:235–41. 10.1111/j.1468-3083.2012.04453.x 22313357

[106] Lacour JP. Carcinogenesis of basal cell carcinomas: genetics and molecular mechanisms. Br J Dermatol. (2002) 61:17–9. 10.1046/j.1365-2133.146.s61.5.x 11966727

[107] Daya-Grosjean L Sarasin A. UV-specific mutations of the human patched gene in basal cell carcinomas from normal individuals and xeroderma pigmentosum patients. Mutat Res. (2000) 450:193–9. 10.1016/S0027-5107(00)00025-7 10838143

[108] Kim M Park H Baek S Byun D Houh D. Mutations of the p53 and PTCH gene in basal cell carcinomas: UV mutation signature and strand bias. J Dermatol Sci. (2002) 29:1–9. 10.1016/S0923-1811(01)00170-0 12007715

[109] Atwood S Sarin K Li J Yao C Urman N Chang A. Rolling the genetic dice: neutral and deleterious smoothened mutations in drug-resistant basal cell carcinoma. The J Invest Dermatol. (2015) 135:2138–41. 10.1038/jid.2015.115 25801792 PMC4504757

[110] Xie J Murone M Luoh S Ryan A QM G CH Z. Activating smoothened mutations in sporadic basal-cell carcinoma. Nature. (1998) 391:90–2. 10.1038/34201 9422511

[111] Urman NM Mirza A Atwood SX Whitson RJ Oro AE. Tumor-derived suppressor of fused mutations reveal hedgehog pathway interactions. PLoS ONE. (2016) 11:e168031. 10.1371/journal.pone.0168031 28030567 PMC5193403

[112] Ian S Narang MA Tim E Cornelia H Yusuke N Georgia CT. Isolation and Characterization of Human Patched 2 (PTCH2), a putative tumour suppressor gene in basal cell carcinoma and medulloblastoma on chromosome 1p32. Hum Mol Genet. (1999) 8:291–7. 10.1093/hmg/8.2.291 9931336

[113] Zwaan S Haass N. Genetics of basal cell carcinoma. Australas J Dermatol. (2010) 51:81–92; quiz 93–4. 10.1111/j.1440-0960.2009.00579.x 20546211

[114] Aubrey BJ Strasser A Kelly GL. Tumor-suppressor functions of the TP53 pathway. Cold Spring Harb Perspect Med. (2016) 6:a26062. 10.1101/cshperspect.a026062 PMC4852799 27141080

[115] Benjamin CL Ananthaswamy HN. p53 and the pathogenesis of skin cancer. Toxicol Appl Pharmacol. (2007) 224:241–8. 10.1016/j.taap.2006.12.006 17270229 PMC2080850

[116] Giglia-Mari G Sarasin A. TP53 mutations in human skin cancers. Hum Mutat. (2010) 21:217–28. 10.1002/humu.10179 12619107

[117] Rosenstien BS Phelps RG Weinstock MA Bernstein JL Gordon ML Rudikoff D. p53 Mutations in Basal Cell Carcinomas Arising in Routine Users of Sunscreens. Photochem Photobiol. (2010) 70:798–806. 10.1111/j.1751-1097.1999.tb08285.x 10568172

[118] Rachakonda PS Hosen I de Verdier P Fallah M Heidenreich B Ryk C. TERT promoter mutations in bladder cancer affect patient survival and disease recurrence through modification by a common polymorphism. Proc Natl Acad Sci U S A. (2013) 110:17426–31. 10.1073/pnas.1310522110 24101484 PMC3808633

[119] Heidenreich B Rachakonda PS Hemminki K Kumar R. TERT promoter mutations in cancer development. Curr Opin Genet Dev. (2014) 24:30–7. 10.1016/j.gde.2013.11.005 24657534

[120] Scott GA Laughlin TS Rothberg PG. Mutations of the TERT promoter are common in basal cell carcinoma and squamous cell carcinoma. Mod Pathol. (2014) 27:516–23. 10.1038/modpathol.2013.167 24030752

[121] Griewank KG Murali R Schilling B Schimming T Möller I Moll I. TERT promoter mutations are frequent in cutaneous basal cell carcinoma and squamous cell carcinoma. PLoS ONE. (2013) 8:e80354. 10.1371/journal.pone.0080354 24260374 PMC3832433

[122] Pópulo H Boaventura P Vinagre J Batista R Mendes A Caldas R. TERT promoter mutations in skin cancer: the effects of sun exposure and X-irradiation. J Invest Dermatol. (2014) 134:2251–7. 10.1038/jid.2014.163 24691053

[123] Wang L Shi Y Ju P Liu R Yeo SP Xia Y. Silencing of diphthamide synthesis 3 (Dph3) reduces metastasis of murine melanoma. PLoS ONE. (2012) 7:e49988. 10.1371/journal.pone.0049988 23185508 PMC3502187

[124] Denisova E Heidenreich B Nagore E Rachakonda PS Hosen I Akrap I. Frequent DPH3 promoter mutations in skin cancers. Oncotarget. (2015) 6:35922–30. 10.18632/oncotarget.5771 26416425 PMC4742151

[125] Rivas JM Ullrich SE. The role of IL-4, IL-10, and TNF-alpha in the immune suppression induced by ultraviolet radiation. J Leukoc Biol. (1994) 56:769–75. 10.1002/jlb.56.6.769 7996051

[126] Mukhtar H Elmets CA. Photocarcinogenesis: mechanisms, models and human health implications: introduction. Photochem Photobiol. (1996) 63:356–7. 10.1111/j.1751-1097.1996.tb03040.x 8934734

[127] Katiyar SK Meeran SM. Obesity increases the risk of UV radiation-induced oxidative stress and activation of MAPK and NF-κB signaling. Free Radic Biol Med. (2007) 42:299–310. 10.1016/j.freeradbiomed.2006.10.049 17189835 PMC1805635

[128] Gilmore TD. Introduction to NF-kappaB: players, pathways, perspectives. Oncogene. (2006) 25:6680–4. 10.1038/sj.onc.1209954 17072321

[129] Bargou RC Emmerich F Krappmann D Bommert K Dörken B. Constitutive nuclear factor-kappaB-RelA activation is required for proliferation and survival of Hodgkin's disease tumor cells. J Clin Invest. (1997) 100:2961–9. 10.1172/JCI119849 9399941 PMC508507

[130] Duffey DC Chen Z Dong G Ondrey FG Wolf J Brown K. Expression of a dominant-negative mutant inhibitor-kBa of nuclear factor-k Bi n human head and neck squamous cell carcinoma inhibits survival, proinflammatory cytokine expression, and tumor growth *in vivo*. Cancer Res. (1999) 59:3468–74. 10416612

[131] Poppelmann B Klimmek K Strozyk E Voss R Schwarz T Kulms D. NF{kappa}B-dependent down-regulation of tumor necrosis factor receptor-associated proteins contributes to interleukin-1-mediated enhancement of ultraviolet B-induced apoptosis. J Biol Chem. (2005) 280:15635–43. 10.1074/jbc.M413006200 15723831

[132] Lu W Laszlo C Miao Z Chen H Wu S. the role of nitric-oxide synthase in the regulation of UVB light-induced phosphorylation of the subunit of eukaryotic initiation factor 2. J Biol Chem. (2009) 284:24281–8. 10.1074/jbc.M109.008821 19586904 PMC2782021

[133] Weng H Deng Y Xie Y Liu H Gong F. Expression and significance of HMGB1, TLR4 and NF-κB p65 in human epidermal tumors. BMC Cancer. (2013) 13:311. 10.1186/1471-2407-13-311 23803172 PMC3697986

[134] Tong L Wu S. The role of constitutive nitric oxide synthase in ultraviolet B light-induced nuclear factor kappa B activity. J Biol Chem. (2014) 289:26658–68. 10.1074/jbc.M114.600023 25112869 PMC4176213

[135] Johnson K Wulff B Oberyszyn T Wilgus T. Ultraviolet light exposure stimulates HMGB1 release by keratinocytes. Arch Dermatol Res. (2013) 305:805–15. 10.1007/s00403-013-1401-2 23942756 PMC3818797

[136] Nguyen AH Detty SQ Agrawal DK. Clinical Implications of High-mobility Group Box-1 (HMGB1) and the Receptor for Advanced Glycation End-products (RAGE) in cutaneous malignancy: a systematic review. Anticancer Res. (2017) 37:1. 10.21873/anticanres.11282 28011467

[137] Harberts E Gaspari A. TLR signaling and DNA repair: are they associated? J Invest Dermatol. (2012) 133:296–302. 10.1038/jid.2012.288 22931928 PMC8381358

[138] Russo I Cona C Saponeri A Bassetto F Baldo V Alaibac M. Association between toll-like receptor 7 gln11leu single-nucleotide polymorphism and basal cell carcinoma. Biomed Rep. (2016) 4:459–62. 10.3892/br.2016.597 27073632 PMC4812152

[139] Stockfleth E Trefzer U Garcia-Bartels C Wegner T Sterry W. The use of Toll-like receptor-7 agonist in the treatment of basal cell carcinoma: an overview. Br J Dermatol. (2003) 149 (Suppl. 66):53–6. 10.1046/j.0366-077X.2003.05626.x 14616352

[140] Fishelevich R Zhao Y Tuchinda P Liu H Nakazono A Tammaro A. Imiquimod-induced TLR7 signaling enhances repair of DNA damage induced by ultraviolet light in bone marrow-derived cells. J Immunol. (2011) 187:1664. 10.4049/jimmunol.1100755 21765012 PMC3150393

[141] Madson J Hansen L. Multiple mechanisms of Erbb2 action after ultraviolet irradiation of the skin. Mol Carcinogen. (2007) 46:624–8. 10.1002/mc.20335 17477367

[142] Stephens P Hunter C Gignell G Edkins S Davies H Teague J. Lung cancer: intragenic ERBB2 kinase mutations in tumours. Nature. (2004) 431:525–6. 10.1038/431525b 15457249

[143] Rao VH Vogel K Yanagida JK Marwaha N Kandel A Trempus C. Erbb2 up-regulation of ADAM12 expression accelerates skin cancer progression. Mol Carcinog. (2015) 54:1026. 10.1002/mc.22171 24798404

[144] Franchi L Mcdonald C Kanneganti TD Amer A Nunez G. Nucleotide-binding oligomerization domain-like receptors: intracellular pattern recognition molecules for pathogen detection and host defense. J Immunol. (2006) 177:3507–13. 10.4049/jimmunol.177.6.3507 16951308

[145] Martinon F Mayor A Tschopp J. The inflammasomes: guardians of the body. Annu Rev Immunol. (2009) 27:229–65. 10.1146/annurev.immunol.021908.132715 19302040

[146] Lamkanfi M. Emerging inflammasome effector mechanisms. Nat Rev Immunol. (2011) 11:213–20. 10.1038/nri2936 21350580

[147] Ahmad I Muneer KM Chang ME Nasr HM Clay JM Huang CC. Ultraviolet radiation-induced downregulation of SERCA2 mediates activation of NLRP3 inflammasome in basal cell carcinoma. Photochem Photobiol. (2017) 93:1025–33. 10.1111/php.12725 28120514

[148] Zhang J Bowden GT. UVB irradiation regulates Cox-2 mRNA stability through AMPK and HuR in human keratinocytes. Mol Carcinogen. (2010) 47:974–83. 10.1002/mc.20450 18449856

[149] Tim Bowden G. Prevention of non-melanoma skin cancer by targeting ultraviolet-B-light signalling. Nat Rev Cancer. (2004) 4:23–35. 10.1038/nrc1253 14681688

[150] Liu K Yu D Cho YY Bode AM Ma W Yao K. Sunlight UV-induced skin cancer relies upon activation of the p38 signaling pathway. Cancer Res. (2013) 73:2181–8. 10.1158/0008-5472.CAN-12-3408 23382047 PMC3618616

[151] Segrelles C Ruiz S Perez P Murga C Santos M Budunova IV. Functional roles of Akt signaling in mouse skin tumorigenesis. Oncogene. (2002) 21:53–64. 10.1038/sj.onc.1205032 11791176

[152] Segrelles C Lu J Hammann B Santos M Di Giovanni J. Deregulated activity of Akt in epithelial basal cells induces spontaneous tumors and heightened sensitivity to skin carcinogenesis. Cancer Res. (2007) 67:10879–88. 10.1158/0008-5472.CAN-07-2564 18006833

[153] Wu CL Qiang L Han W Ming M He YY. Role of AMPK in UVB-induced DNA damage repair and growth control. Oncogene. (2012) 32:2682–9. 10.1038/onc.2012.279 22751115 PMC3465498

[154] Fischer SM Lo HH Gordon GB Seibert K Kelloff G Lubet RA. Chemopreventive activity of celecoxib, a specific cyclooxygenase-2 inhibitor, and indomethacin against ultraviolet light-induced skin carcinogenesis. Mol Carcinogen. (2015) 25:231–40. 10.1002/(SICI)1098-2744(199908)25:4&lt;231::AID-MC1&gt;3.0.CO;2-F 10449029

[155] Rundhaug JE Mikulec C Pavone A Fischer SM. A role for cyclooxygenase-2 in ultraviolet light-induced skin carcinogenesis. Mol Carcinogen. (2007) 46:692–8. 10.1002/mc.20329 17443745

[156] Wilgus TA Koki AT Zweifel BS Rubal PA Oberyszyn TM. Chemotherapeutic efficacy of topical celecoxib in a murine model of ultraviolet light B-induced skin cancer. Mol Carcinog. (2003) 38:33–9. 10.1002/mc.10142 12949841

[157] Mikulec CD Rundhaug JE Simper MS Lubet RA Fischer SM. The chemopreventive efficacies of nonsteroidal anti-inflammatory drugs: the relationship of short-term biomarkers to long-term skin tumor outcome. Cancer Prev Res. (2013) 6:675–85. 10.1158/1940-6207.CAPR-13-0064 23682071 PMC3701752

[158] Robertson MJ Ritz J. Interleukin 12: basic biology and potential applications in cancer treatment. Oncologist. (1996) 1:88–97. 10.1634/theoncologist.1-1-88 10387973

[159] Meeran SM Katiyar S Elmets CA Katiyar SK. Interleukin-12 deficiency is permissive for angiogenesis in UV radiation-induced skin tumors. Cancer Res. (2007) 67:3785–93. 10.1158/0008-5472.CAN-06-3134 17440092 PMC1986731

[160] Katiyar SK. Interleukin-12 and photocarcinogenesis. Toxicol Appl Pharmacol. (2007) 224:220–7. 10.1016/j.taap.2006.11.017 17239911 PMC2080793

[161] Schwarz A Ständer S Berneburg M Böhm M Kulms D van Steeg H. Interleukin-12 suppresses ultraviolet radiation-induced apoptosis by inducing DNA repair. Nat Cell Biol. (2002) 4:26–31. 10.1038/ncb717 11780128

[162] Meeran SM Punathil T Katiyar SK. IL-12 deficiency exacerbates inflammatory responses in UV-irradiated skin and skin tumors. J Invest Dermatol. (2008) 128:2716–27. 10.1038/jid.2008.140 18509359 PMC2574589

